# Hepatic Inflammation and Apoptosis in Socially Isolated Rats: Effects of Vortioxetine Treatment

**DOI:** 10.5812/ijpr-170887

**Published:** 2026-06-15

**Authors:** Neziha Senem Arı, Merve Akkuş, Hatice Solak, Raviye Özen Koca, Işık Solak Görmüş

**Affiliations:** 1 Department of Histology and Embryology, Faculty of Medicine, Kutahya Health Sciences University, Kütahya, Turkey; 2 Department of Psychiatry, Faculty of Medicine, Kutahya Health Sciences University, Kütahya, Turkey; 3 Department of Physiology, Faculty of Medicine, Kutahya Health Sciences University, Kütahya, Turkey; 4 Department of Physiology, Meram Faculty of Medicine, Necmettin Erbakan University, Konya, Turkey

**Keywords:** Social Isolation, Vortioxetine, Hepatic Inflammation, Apoptosis

## Abstract

**Background:**

Depression and chronic psychosocial stress can induce systemic inflammation and oxidative stress that may extend beyond the central nervous system to peripheral organs such as the liver. Social isolation is a widely used rodent model in depression research; however, its hepatic consequences and the potential protective effects of antidepressant treatment remain incompletely characterized.

**Objectives:**

This study aimed to investigate whether social isolation induces hepatic inflammation and apoptosis in rats and to evaluate the effects of vortioxetine treatment on these hepatic alterations.

**Methods:**

Forty male Wistar albino rats were randomly allocated into four groups (n = 10/group): Control (C), Social Isolation (SI), Vortioxetine (V), and Social Isolation + Vortioxetine (SI+V). Social isolation was maintained for four weeks. Vortioxetine (10 mg/kg/day, intraperitoneally) or vehicle (dimethyl sulfoxide, 1 mL/kg) was administered from days 17 to 31. Liver tissues were examined histopathologically using hematoxylin-eosin and Masson's trichrome staining. Apoptosis was assessed using a terminal deoxynucleotidyl transferase dUTP nick-end labeling assay and caspase-3 immunoreactivity. Immunohistochemical analyses were performed for tumor necrosis factor-alpha, nuclear factor-kappa B p65, and cytokeratin-18. Hepatic interleukin-6 levels were quantified by enzyme-linked immunosorbent assay.

**Results:**

The SI group exhibited marked sinusoidal dilatation, erythrocyte extravasation, hepatocellular ballooning, and inflammatory infiltration, accompanied by increased tumor necrosis factor-alpha, nuclear factor-kappa B p65, and caspase-3 immunoreactivity; increased terminal deoxynucleotidyl transferase dUTP nick-end labeling positivity; and elevated hepatic interleukin-6 levels (P < 0.001). Vortioxetine treatment in isolated rats attenuated these inflammatory and apoptotic responses, resulting in lower histopathological damage scores and better cytokeratin-18 preservation than in the SI group. Masson's trichrome staining showed no significant collagen accumulation among groups (P > 0.05).

**Conclusions:**

Social isolation was associated with increased hepatic inflammation and apoptosis, as demonstrated by histopathological, immunohistochemical, and biochemical findings. Vortioxetine treatment was associated with partial attenuation of these alterations. These findings are limited to the measured endpoints and do not establish direct functional effects.

## 1. Background

Depressive disorders, including major depressive disorder and dysthymia, constitute a substantial global public health burden. According to the Global Burden of Disease 2021 estimates, depressive disorders are among the leading causes of years lived with disability and contribute substantially to the overall disability-adjusted life-year burden. Disability-adjusted life-years represent the sum of years of life lost due to premature death and years lived with disability, providing a quantitative measure of overall population health loss (1, 2).

In experimental research, the social isolation model is frequently used to elucidate the biological mechanisms of depressive disorders and to evaluate potential therapeutic approaches (3). Chronic social isolation is a well-established psychosocial stress paradigm that has been reported to elicit depression- and anxiety-like behavioral alterations in rodents, including anhedonia-related changes, and to dysregulate stress-responsive neuroendocrine systems. Beyond its central effects, social isolation has also been associated with systemic low-grade inflammation and oxidative stress, providing a mechanistic link between stress-related neurobiology and peripheral organ vulnerability, including liver vulnerability (3, 4). In rodents, social isolation maintained for 4 weeks (28 days) has been reported to disrupt hypothalamic-pituitary-adrenal axis function, a key stress response system, thereby adversely affecting the immune, digestive, and nervous systems (4). Moreover, chronic stress has been reported to impair not only emotional regulation but also peripheral organs, particularly the liver, through oxidative stress and inflammation (5). Animal studies have shown that chronic stress elevates interleukin-6 (IL-6), tumor necrosis factor-alpha (TNF-alpha), and interleukin-1 beta expression (6). These proinflammatory cytokines activate the Janus kinase 2/signal transducer and activator of transcription 3 signaling pathway, aggravate hepatic inflammation, induce hepatocyte necrosis and apoptosis, and consequently increase serum transaminase levels (7). Furthermore, a bidirectional relationship has been reported between depression and nonalcoholic fatty liver disease, with both conditions linked to systemic inflammatory processes (8). Consistently, rat models exposed to social isolation and chronic stress have demonstrated increased hepatic inflammation and oxidative stress markers, as well as elevated serum alanine aminotransferase and aspartate aminotransferase levels (9, 10).

Vortioxetine is a multimodal serotonergic antidepressant approved for major depressive disorder. It exerts its pharmacological effects through antagonism of 5-hydroxytryptamine 3 and 5-hydroxytryptamine 7 receptors, agonist activation of 5-hydroxytryptamine 1A receptors, and partial agonism of 5-hydroxytryptamine 1B receptors (11). Both preclinical and clinical studies have demonstrated that vortioxetine is effective and well tolerated in alleviating symptoms commonly associated with major depressive disorder, such as depression, anxiety, stress, and cognitive dysfunction (12). Although the central nervous system effects of vortioxetine are well established, data on its potential effects on peripheral organs, such as the liver, remain limited. Recent experimental findings suggest that vortioxetine administration may influence markers of inflammation, oxidative stress, and cellular damage in hepatic tissue (13). Given the intersection among serotonergic modulation, the stress response, and immune regulation, vortioxetine may modulate hepatic inflammation and apoptosis in social isolation-associated conditions relevant to depression.

## 2. Objectives

Existing literature indicates that social isolation can induce structural and functional impairments in the liver, whereas vortioxetine treatment may alter liver enzyme activity, histological architecture, and inflammatory responses under various conditions. To date, the combined influence of these 2 factors on hepatic tissue has not been systematically evaluated at the histopathological and immunohistochemical levels. Therefore, this study aimed to evaluate the effects of vortioxetine treatment on structural and cellular alterations in the liver tissue of rats exposed to social isolation using histopathological and immunohistochemical analyses targeting inflammatory and apoptotic markers. This approach is expected to provide a more integrative and comprehensive understanding of the interaction between disease- and treatment-related changes in hepatic tissue than that provided by previous studies.

## 3. Methods

### 3.1. Experimental Animals, Ethics, and Housing Conditions

Male Wistar albino rats aged > 4 months and weighing 300 - 400 g were used. The animals were housed under controlled environmental conditions (temperature, 24 ± 2°C; relative humidity, 55 ± 15%; 12-hour light/dark cycle). Standard pellet chow and tap water were provided ad libitum. Animals were housed in standard polycarbonate cages with wood shaving-based bedding routinely used in the institutional animal facility. Although the specific commercial type of bedding was not formally recorded, it was changed regularly according to standard housing procedures. Environmental enrichment was not provided to maintain consistency across experimental conditions. All procedures were performed in accordance with national legislation and international ethical standards. The experiment was conducted at the Laboratory Animal Research and Breeding Center and the Department of Histology and Embryology, Faculty of Medicine, Kütahya Health Sciences University (KSBÜ), with ethical approval from the Local Animal Experiments Ethics Committee of KSBÜ (Approval No. 2025.09.03; date: 21.08.2025). Animals were monitored daily for general health status and signs of distress, and no humane endpoints were reached during the study.

Animals were housed in standard polycarbonate cages measuring approximately 40 × 25 × 20 cm. Animals were handled once daily during routine monitoring and injection procedures. For isolated animals, visual and physical contact was prevented, and cages were positioned to minimize direct visual exposure; however, limited olfactory cues may have persisted within the same room environment.

### 3.2. Experimental Design, Social Isolation Model, and Vortioxetine Administration

Rats were randomly assigned to 4 experimental groups (n = 10 per group; total n = 40):

1) Control (C): Animals were maintained under standard cage conditions for 4 weeks (28 days). Beginning on day 17 (days 17 - 31, 15 consecutive days), they received once-daily intraperitoneal injections of the vehicle dimethyl sulfoxide (DMSO; 10% in 0.9% NaCl; 1 mL/kg).

2) Social Isolation (SI): Animals were housed individually to induce social isolation for 4 weeks (28 days) and received the same vehicle injections (DMSO; 10% in 0.9% NaCl; 1 mL/kg, intraperitoneally) during the last 15 days (days 17 - 31). Animals had no visual or physical contact; however, limited olfactory cues may have been present due to shared room conditions.

3) Vortioxetine (V): Animals were maintained under standard cage conditions for 4 weeks (28 days) and received vortioxetine hydrobromide (Sigma-Aldrich, St. Louis, MO, USA; Cat. No. SML3388; CAS No. 960203 - 27 - 4) at 10 mg/kg/day intraperitoneally for 15 consecutive days beginning on day 17 (days 17 - 31), following the protocol of Bétry et al. (14). The initiation of vortioxetine treatment on day 17 was intended to allow the establishment of social isolation-induced stress-related alterations before pharmacological intervention, as commonly applied in similar experimental paradigms.

4) Social Isolation + Vortioxetine (SI+V): Animals were subjected to 4 weeks (28 days) of social isolation and treated with vortioxetine hydrobromide (10 mg/kg/day, intraperitoneally) during the last 15 days (days 17 - 31) (14).

For the social isolation model, rats were housed individually in cages covered with an opaque black gelatin film, preventing visual and tactile contact between animals and minimizing exposure to external stimuli. The isolation period lasted 4 weeks (28 days) (3, 15). Notably, the social isolation setup, including individual housing and opaque cage covering, was maintained throughout the experimental period, including injection days, thereby minimizing unintended social exposure during daily handling. To minimize potential confounding effects of handling, all injections were administered at the same time each day. During the injection procedure, animals were handled one at a time, removed individually to the procedure area, injected within a short, standardized timeframe, and immediately returned to their own cages. This procedure was applied uniformly across all experimental groups to avoid systematic handling-related bias. Vortioxetine was freshly prepared daily and dissolved in 10% DMSO in 0.9% NaCl. The injection volume was standardized to 1 mL/kg body weight. All injections were administered at the same time each day to minimize circadian variability.

### 3.3. Sample Size Justification

The sample size (n = 10 per group) was determined based on prior experimental studies in similar rodent models, in which comparable group sizes were sufficient to detect statistically significant differences in inflammatory and apoptotic parameters. Although a formal a priori power analysis was not performed because of the exploratory nature of the study, the observed effect sizes for key outcomes were large, indicating that the study had adequate statistical power to detect biologically relevant differences.

The experimental unit was defined as the individual animal. For each parameter, multiple measurements were averaged per animal to avoid pseudo-replication. This approach is consistent with ARRIVE guidelines for transparent reporting of experimental design and sample size justification in animal studies.

### 3.4. Randomization and Blinding

Animals were randomly allocated to experimental groups using a computer-generated randomization sequence by an investigator not involved in outcome assessment. Allocation was concealed until the start of the experimental procedures.

All outcome assessments were performed by investigators blinded to group allocation. Histopathological scoring, immunohistochemical evaluation using H-scores, and terminal deoxynucleotidyl transferase dUTP nick-end labeling (TUNEL) quantification were conducted independently by 2 blinded observers, and the mean of their evaluations was used for analysis. All experimental procedures, outcome definitions, and analysis plans were predefined before data collection to ensure methodological transparency and reproducibility.

### 3.5. Tissue Collection and Processing

After completion of the experimental period, deep anesthesia was induced by intramuscular injection of ketamine (75 mg/kg) and xylazine (10 mg/kg). Under full anesthesia, animals were decapitated, and liver tissues were rapidly excised. A portion of each liver was fixed in 10% neutral-buffered formalin for 48 hours for histopathological examination. The remaining samples, intended for enzyme-linked immunosorbent assay (ELISA), were immediately frozen and stored at -80°C until use.

All animals completed the experimental protocol. No animals or samples were excluded from the study, and all collected data were included in the final analysis.

### 3.6. Histological Staining

#### 3.6.1. Hematoxylin-Eosin and Masson's Trichrome Protocols

Fixed liver tissues were processed routinely, embedded in paraffin, and sectioned at 4 µm thickness. Sections were incubated at 60°C for 60 minutes, deparaffinized in xylene (3 × 10 minutes), rehydrated through graded alcohols (100%, 96%, 90%, and 70%), and rinsed with distilled water.

For hematoxylin-eosin (H&E) staining, sections were stained with Harris hematoxylin for 2 minutes, rinsed, briefly differentiated in 1% ammonia water, washed again, and counterstained with eosin for 2 minutes. Sections were dehydrated through ascending alcohols, followed by clearing in xylene (2 × 1 minute) and mounting with Entellan.

For Masson's trichrome staining, after the H&E pretreatment steps, sections were stained sequentially with Weigert's iron hematoxylin (5 minutes), picric acid (5 minutes), Biebrich scarlet-acid fuchsin (5 minutes), phosphotungstic/phosphomolybdic acid (5 minutes), and aniline blue (5 minutes). Slides were dehydrated, cleared in xylene, and mounted with Entellan (Masson's Trichrome Stain Kit; Bio-Optica, Cat. No. 04 - 010802).

Slides were examined and photographed using a Zeiss Axiocam 208 color light microscope.

#### 3.6.2. Histopathological Scoring

All histopathological evaluations were performed in a blinded manner by 2 independent observers. Discrepancies between observers were resolved by consensus. For each animal, 5 non-overlapping fields were evaluated, and the mean score was calculated as a single representative value per animal.

### 3.6.2.1. Modified Liver Parenchymal Injury Score (0 - 3)

Grade 0: No or minimal damage; hepatocyte morphology preserved and cytoplasm normal.

Grade 1 (mild): Focal nuclear pyknosis with slightly pale cytoplasm; small cytoplasmic vacuoles may be observed. Cell borders are intact.

Grade 2 (moderate): Patchy or widespread nuclear pyknosis, pale or swollen cytoplasm consistent with ballooning/hydropic degeneration, and partial loss of cell border integrity; moderate vacuolization may accompany these changes.

Grade 3 (severe): Diffuse injury; disruption of hepatic cord architecture, extensive cytoplasmic swelling/pallor, and marked vacuolization, frequently associated with inflammatory cell infiltration.

This scoring system was adapted to better reflect the predominant morphological pattern in this study (16). The criterion of cytoplasmic hypereosinophilia was replaced with the presence of pale or vacuolated cytoplasm, representing ballooning/hydropic degeneration, while necrosis was excluded. Five random fields from the portal, midzonal, and perivenular regions at 20× magnification were evaluated per animal, and the mean score was recorded as the parenchymal injury index.

### 3.6.2.2. Microcirculatory (Sinusoidal) Injury Scoring (0 - 3)

Sinusoidal dilatation and erythrocyte extravasation were assessed separately on H&E-stained sections in a blinded manner. Five random fields from the portal, midzonal, and perivenular regions at 20× magnification were analyzed per slide.

Dilatation was scored based on lumen width and cord spacing as follows: 0 = normal, 1 = focal (< 10%), 2 = patchy (10% - 30%), and 3 = diffuse (> 30%).

Erythrocyte extravasation was scored according to the extent of extravasated erythrocytes as follows: 0 = absent, 1 = focal (< 10%), 2 = patchy (10% - 30%), and 3 = diffuse (> 30%).

### 3.6.2.3. Masson's Trichrome Evaluation

Digital images were analyzed using Fiji/ImageJ (v1.x) software to calculate the percentage of collagen area. At least 5 random fields per sample were evaluated, and the mean percentage was used for group comparisons. Statistical significance was set at P < 0.05.

### 3.7. Apoptosis Analysis by TUNEL Staining

Cell counting was performed manually by a blinded observer.

#### 3.7.1. Section Preparation and Protocol

Paraffin-embedded sections (4 µm) were incubated at 60°C for 1 hour, deparaffinized in xylene (3 × 5 minutes), and rehydrated through descending alcohols (100%, 96%, 90%, and 70%). After incubation with proteinase K at 37°C for 15 minutes, endogenous peroxidase activity was blocked using 3% H_2_O_2_ for 5 minutes.

#### 3.7.2. Labeling and Detection

Apoptotic cells were detected using the ApopTag Peroxidase In Situ Apoptosis Detection Kit (Millipore/Merck, Darmstadt, Germany; Cat. No. S7101) according to the manufacturer's instructions. The procedure included labeling of DNA 3'-OH ends by terminal deoxynucleotidyl transferase, detection with anti-digoxigenin horseradish peroxidase, and visualization using 3,3'-diaminobenzidine chromogen. Sections were lightly counterstained with hematoxylin.

#### 3.7.3. Controls and Quantification

Sections treated with DNase I served as positive controls, while those processed without terminal deoxynucleotidyl transferase enzyme served as negative controls. Apoptotic nuclei were identified by brown 3,3'-diaminobenzidine nuclear staining; cytoplasmic staining was not counted. Ten random perivenular fields at 200× magnification were analyzed per section.

The TUNEL index (%) was calculated as follows:

$$\mathrm{TUNEL}\mathrm{Index}(\%)=(\frac{\mathrm{Number}\mathrm{of}\mathrm{TUNEL}-\mathrm{positive}\mathrm{hepatocyte}\mathrm{nuclei}}{\mathrm{Total}\mathrm{number}\mathrm{of}\mathrm{hepatocyte}\mathrm{nuclei}})\times 100$$

### 3.8. Immunohistochemistry

#### 3.8.1. Section Preparation and Antigen Retrieval

Paraffin sections (4 µm) were incubated at 60°C for 1 hour, deparaffinized in xylene (3 × 5 minutes), and rehydrated through graded alcohols. Antigen retrieval was performed using 1/10 diluted citrate buffer (pH 6.0, AP-9003 - 999; Thermo Scientific) in a PT Module (A80400012; LabVision).

#### 3.8.2. Blocking and Primary Antibodies

Endogenous peroxidase activity was quenched with 3% H_2_O_2_ for 10 minutes, followed by blocking with Protein Block (TA-125-PBQ; Thermo Scientific) for 10 minutes. Sections were then incubated with the following primary antibodies for 2 hours at room temperature: TNF-alpha (Proteintech, Rosemont, IL, USA; Cat. No. 60291 - 1-IG; 1:200), nuclear factor-kappa B (NF-kappa B) p65 (Boster, Pleasanton, CA, USA; Cat. No. PA1669; 1:200), caspase-3 (pan/total) (Thermo Scientific, Fremont, CA, USA; Cat. No. RB-1197-P0; 1:100), and cytokeratin-18 (CK-18) (pan/total) (Biorbyt, Cambridge, UK; Cat. No. orb500815; 1:100).

#### 3.8.3. Secondary Antibody and Chromogen Development

Signal amplification was performed using Amplifier Quanto (TL-125-QPB; 20 minutes) and horseradish peroxidase Polymer Quanto (TL-125-QPH; 30 minutes), with phosphate-buffered saline rinses between steps. Immunoreactivity was visualized using 3,3'-diaminobenzidine chromogen (TA-125-HA; Thermo Scientific), followed by counterstaining with hematoxylin. In some sections, 3-amino-9-ethylcarbazole chromogen (TA-125-UG; Thermo Scientific) was used, and slides were coverslipped with an aqueous mounting medium. All immunostaining procedures were performed on a Sequenza Immunostaining Center (Shandon/Thermo Scientific; Cat. No. 73300001). Secondary antibody polymers were supplied in a ready-to-use format and were applied without additional dilution.

### 3.9. Immunohistochemical Evaluation

Immunohistochemical scoring was performed by blinded observers unaware of group allocation. Fields were selected randomly within the perivenular region using a systematic sampling approach.

#### 3.9.1. H-Score Method

The cytoplasmic immunoreactivity of TNF-alpha, NF-kappa B p65, and caspase-3 was evaluated semiquantitatively using the H-score method. Because staining was confined to perivenular regions, analyses were restricted to these areas. Ten random, non-overlapping fields at 200× magnification were evaluated per section. Staining intensity was graded as 0 (negative), 1 (weak), 2 (moderate), and 3 (strong). The H-score ranged from 0 to 300 and was calculated as follows: H-score = Σ (Pi × i) where Pi represents the percentage of cells stained at each intensity level (i = 0 - 3), and ΣPi = 100.

#### 3.9.2. CK-18 Perivenular Staining Score (0 - 3)

Perivenular CK-18 staining preservation was scored as follows: 0 = intact, diffuse staining; 1 = slightly reduced staining, with focal pale areas; 2 = moderately reduced staining, with patchy discontinuities; and 3 = markedly decreased or lost staining, with diffuse weak staining or extensive loss. Ten random perivenular fields at 200× magnification were evaluated blindly for each case.

### 3.10. ELISA Analysis

Liver tissues were homogenized according to the manufacturer's protocol provided with the ELISA kit. IL-6 protein levels were quantified using a commercial ELISA kit (Bioassay Technology Laboratory, BT LAB, Shanghai, China; Cat. No. E0135Ra). All tissue homogenates were analyzed simultaneously under identical laboratory conditions by the same operator. Results were expressed in ng/L.

### 3.11. Statistical Analysis

All statistical analyses were performed using IBM SPSS Statistics version 27 (IBM Corp., USA). Data normality was assessed using the Shapiro-Wilk test.

The experimental unit for all analyses was the individual animal. For each parameter, measurements obtained from multiple fields or sections were averaged to yield a single value per animal, thereby avoiding pseudo-replication.

For nonnormally distributed continuous variables, including IL-6 levels; TNF-alpha, NF-kappa B p65, and caspase-3 H-scores; TUNEL index; and collagen-positive area percentages, intergroup comparisons were performed using the Kruskal-Wallis test. When a significant overall difference was detected, pairwise comparisons were conducted using the Mann-Whitney U test with Bonferroni correction. Semiquantitative scoring data, including liver parenchymal injury score, sinusoidal dilatation, erythrocyte extravasation, and CK-18 staining score, were ordinal variables and were analyzed using the Kruskal-Wallis test followed by Bonferroni-corrected Mann-Whitney U tests for pairwise comparisons. Data are presented as mean ± standard deviation (SD) for continuous variables and as frequency and percentage (n, %) for categorical or ordinal variables. Effect sizes were calculated to support interpretation of the magnitude of differences. For nonparametric comparisons, rank-biserial correlation (r) was used as the effect size measure. Confidence intervals for effect sizes were not calculated because of the exploratory nature of the study. A P value < 0.05 was considered statistically significant. Specifically, IL-6 levels; TNF-alpha, NF-kappa B p65, and caspase-3 H-scores; TUNEL index; and collagen percentage were analyzed as continuous variables, whereas liver injury scores, sinusoidal dilatation, erythrocyte extravasation, and CK-18 staining scores were analyzed as ordinal variables.

## 4. Results

### 4.1. Histopathological Findings

**Figure 1.**
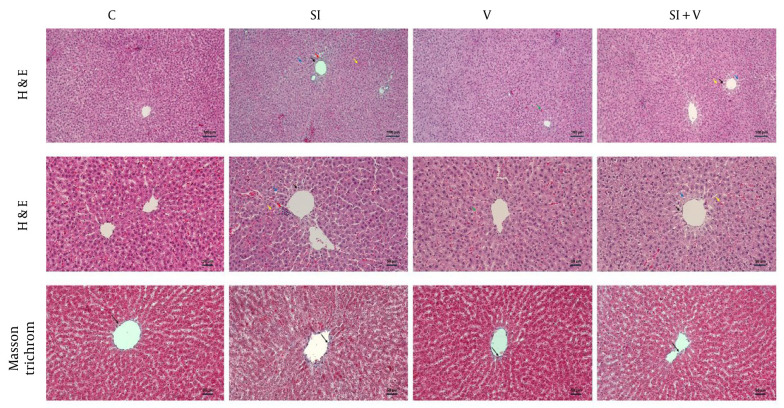
Representative liver sections from the Control (C), Social Isolation (SI), Vortioxetine (V), and Social Isolation + Vortioxetine (SI+V) groups are shown from left to right. The first and second rows display hematoxylin-eosin-stained sections with scale bars of 100 µm and 50 µm, respectively, while the third row shows Masson's trichrome staining with a 50 µm scale bar. Black arrows indicate hepatocytes with pyknotic nuclei, red arrows indicate inflammatory cell infiltration, blue arrows indicate sinusoidal dilatation, yellow arrows indicate erythrocyte extravasation, and long black arrows mark collagen-positive areas. Quantitative analysis of collagen-positive area percentages using Fiji/ImageJ showed no statistically significant differences between groups (Kruskal-Wallis test, P = 0.68). Abbreviations: C, Control; SI, Social Isolation; V, Vortioxetine; SI+V, Social Isolation + Vortioxetine.

Liver architecture was preserved in the C group. In the SI group, hepatocytes with pyknotic nuclei, inflammatory cell infiltration, sinusoidal dilatation, and erythrocyte extravasation were observed in the portal, midzonal, and perivenular regions. In the V group, only mild erythrocyte extravasation was observed. In the SI+V group, these alterations were less pronounced and largely confined to the perivenular region compared with the SI group (Figure 1).

Masson's trichrome analysis showed no significant intergroup difference in collagen deposition (P = 0.68; Figure 1). Liver parenchymal damage scores differed significantly between groups (P < 0.001), with the highest scores in the SI group. Damage was significantly reduced in the SI+V group compared with the SI group, whereas parenchymal integrity was largely preserved in the C and V groups. Post hoc analysis confirmed that the SI group had significantly higher damage scores than the C, V, and SI+V groups, while no significant difference was observed between the C and V groups. Semiquantitative analysis also showed that parenchymal damage, sinusoidal dilatation, and erythrocyte extravasation were significantly higher in the SI group than in the other groups (Table 1).

**Table 1.** Histopathological Liver Parenchymal Damage Scores and Sinusoidal Microcirculatory Findings ^a^

Variables and Grades	C	SI	V	SI + V	Pairwise Comparisons (P Values)
**Parenchymal damage score**					
0	97	3	96	17	SI > C (P < 0.001)
1	3	5	4	59	SI > V (P < 0.001)
2	0	40	0	24	SI > SI+V (P < 0.001)
3	0	52	0	0	C ≈ V (P = 0.527)
**Sinusoidal dilatation score**					
0	95	5	94	10	SI > C (P < 0.001)
1	5	10	6	81	SI > V (P < 0.001)
2	0	35	0	5	SI > SI+V (P < 0.001)
3	0	50	0	4	C ≈ V (P = 0.558)
**Sinusoidal erythrocyte extravasation score**					
0	97	7	84	14	SI > C (P < 0.001)
1	3	61	16	86	SI > V (P < 0.001)
2	0	28	0	0	SI > SI+V (P < 0.001)
3	0	4	0	0	C ≈ V (P = 0.534)

^a^ Abbreviations: C, Control; SI, Social Isolation; V, Vortioxetine; SI+V, Social Isolation + Vortioxetine. Values are expressed as percentage.

### 4.2. Apoptosis and Immunohistochemical Findings

**Figure 2.**
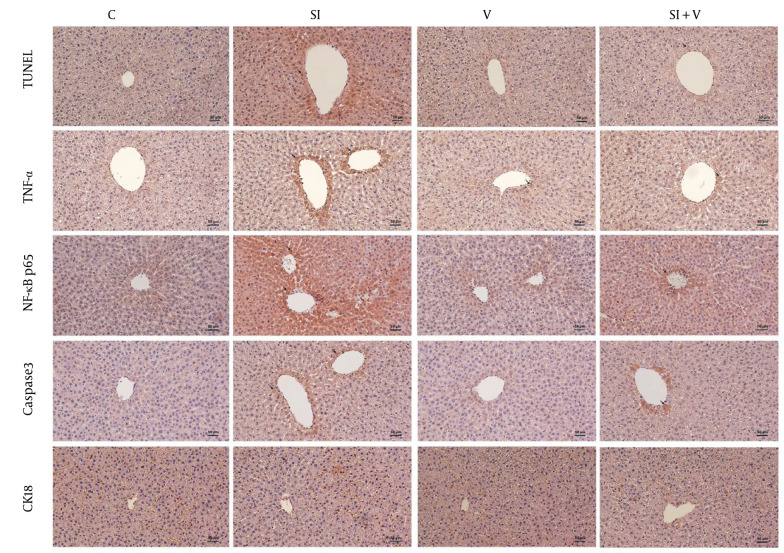
Representative Images of TUNEL and Immunohistochemical Staining for Inflammatory and Apoptotic Markers. Representative micrographs show TUNEL staining (row 1), TNF-alpha (row 2), NF-kappa B p65 (row 3), caspase-3 (row 4), and cytokeratin-18 (row 5) immunoexpression in the C, SI, V, and SI+V groups. All images are from perivenular regions. Increased numbers of TUNEL-positive nuclei and higher immunostaining intensity for TNF-alpha, NF-kappa B p65, and caspase-3 were observed in the SI group compared with the C and V groups. The SI+V group showed a partial reduction in staining compared with the SI group. Cytokeratin-18 staining was reduced in the SI group and partially preserved in the SI+V group (scale bars: 50 µm). Abbreviations: C, Control; SI, Social Isolation; V, Vortioxetine; SI+V, Social Isolation + Vortioxetine.

Apoptotic activity and inflammatory marker expression differed significantly between groups (Table 2 and Figure 2).

**Table 2.** Comparison of TUNEL Index and Immunohistochemical Findings ^a^

Parameters	C	SI	V	SI+V	Pairwise Comparisons (P Values)
**TUNEL index (%)**	1.3 ± 0.02	17.7 ± 4.1	1.5 ± 0.5	5.56 ± 1.4	SI > C (P < 0.001); SI > V (P < 0.001); SI > SI+V (P = 0.029); SI+V > C (P = 0.01); SI+V > V (P = 0.01); C ≈ V (P = 0.298)
**TNF-alpha expression (H-score)**	2.80 ± 0.79	41.10 ± 5.84	3.70 ± 0.95	11.10 ± 2.23	SI > C (P < 0.001); SI > V (P < 0.001); SI > SI+V (P = 0.031); SI+V > C (P = 0.004); SI+V > V (P = 0.009); C ≈ V (P = 0.327)
**NF-kappa B p65 expression (H-score)**	2.90 ± 0.74	158.6 ± 7.73	3.20 ± 0.63	100.67 ± 12.47	SI > C (P < 0.001); SI > V (P < 0.001); SI > SI+V (P = 0.017); SI+V > C (P < 0.001); SI+V > V (P < 0.001); C ≈ V (P = 0.317)
**Caspase-3 expression (H-score)**	0.30 ± 0.48	121.70 ± 7.41	3.40 ± 1.07	65.33 ± 7.87	SI > C (P < 0.001); SI > V (P < 0.001); SI > SI+V (P = 0.024); SI+V > C (P < 0.001); SI+V > V (P < 0.001); C ≈ V (P = 0.330)
**IL-6 (ng/L)**	2.30 ± 0.47	5.76 ± 1.62	2.33 ± 0.83	3.46 ± 1.31	SI > C (P < 0.001); SI > V (P < 0.001); SI > SI+V (P = 0.001); SI+V > C (P = 0.020); SI+V > V (P = 0.023); C ≈ V (P = 0.739)

^a^ Abbreviations: C, Control; IL-6, interleukin-6; NF-kappa B, nuclear factor-kappa B; SI, Social Isolation; SI+V, Social Isolation + Vortioxetine; TNF-alpha, tumor necrosis factor-alpha; V, Vortioxetine. Values are expressed as mean ± SD.

TUNEL Findings: The TUNEL index differed significantly between groups (P < 0.001). The SI group showed the highest levels of apoptosis, whereas the C and V groups showed low and comparable values. Apoptosis was significantly reduced in the SI+V group compared with the SI group. TUNEL positivity was low and similar in the C and V groups (1.3 ± 0.02 and 1.5 ± 0.5, respectively). In the SI+V group, the apoptosis level (5.56 ± 1.4) was significantly lower than that in the SI group, indicating that vortioxetine treatment partially reduced social isolation-induced apoptosis (Table 2).

TNF-alpha Expression: TNF-alpha expression differed significantly between groups (P < 0.001), with the highest levels in the SI group. Expression was low in the C and V groups, whereas the SI+V group showed a significant reduction compared with the SI group (Table 2).

NF-kappa B p65 Expression: NF-kappa B p65 expression was significantly increased in the SI group (P < 0.001). Lower levels were observed in the C and V groups, whereas the SI+V group showed a partial reduction compared with the SI group (Table 2).

Caspase-3 Expression: Caspase-3 expression differed significantly between groups (P < 0.001), with the highest levels in the SI group. Expression was low in the C and V groups, whereas the SI+V group showed reduced levels compared with the SI group (Table 2).

CK-18 Expression: CK-18 staining scores differed significantly between groups (P < 0.001). The SI group showed the greatest loss of staining, whereas the C and V groups showed preserved CK-18 expression. The SI+V group demonstrated partial preservation compared with the SI group (Table 3).

**Table 3.** Distribution of Cytokeratin-18 Perivenular Staining Scores (0 - 3) in Experimental Groups ^a^

Variables and Grades	C	SI	V	SI + V	Pairwise Comparisons (P Values)
**CK-18 staining score**					SI > C (P < 0.001); SI > V (P < 0.001); SI > SI+V (P < 0.001); SI+V > C (P = 0.009); SI+V > V (P = 0.024); C ≈ V (P = 0.534)
0	96	4	91	2	
1	4	5	9	75	
2	0	36	0	23	
3	0	55	0	0	

^a^ Abbreviations: C, Control; CK-18, cytokeratin-18; SI, Social Isolation; SI+V, Social Isolation + Vortioxetine; V, Vortioxetine. Values are expressed as percentage.

### 4.3. Liver IL-6 Protein Levels

Hepatic IL-6 levels differed significantly between groups (P < 0.001). The highest IL-6 concentration was observed in the SI group (5.76 ± 1.62 ng/L), indicating that social isolation triggered a pronounced inflammatory response in liver tissue. IL-6 levels were low and similar in the C (2.30 ± 0.47 ng/L) and V (2.33 ± 0.83 ng/L) groups. In the SI+V group (3.46 ± 1.31 ng/L), a significant decrease was observed compared with the SI group. This finding suggests that vortioxetine treatment partially suppressed the social isolation-associated increase in IL-6. Post hoc pairwise comparisons showed that the SI group had significantly higher IL-6 levels than the C, V, and SI+V groups (P < 0.001 for all), while no significant difference was observed between the C and V groups (P = 0.739) (Table 2).

## 5. Discussion

This study suggests that experimental depression induced by the social isolation model is associated with marked inflammation and apoptosis in the rat liver and that vortioxetine treatment partially alleviates these pathological changes. Histopathological examination revealed sinusoidal dilatation, erythrocyte extravasation, inflammatory cell infiltration, and hepatocyte ballooning in the SI group. TUNEL staining and caspase-3 immunoexpression confirmed increased apoptosis, whereas elevated TNF-alpha and NF-kappa B p65 levels indicated activation of the inflammatory response. Hepatic IL-6 levels were significantly increased in the SI group, and this increase was partially attenuated in the SI+V group. These findings may be related to increased proinflammatory activity and apoptosis-related processes. However, the present findings are based on histopathological and molecular markers rather than direct assessments of liver function. Therefore, the observed effects should be interpreted as indicative of inflammatory and apoptotic alterations rather than as definitive evidence of hepatic dysfunction.

The findings regarding the effects of social isolation on the liver are consistent with the metabolic, inflammatory, and oxidative changes reported in the literature. Social isolation has been reported to increase oxidative stress levels and disrupt glucose homeostasis in rats by impairing hepatic insulin sensitivity (10). In the present study, increased IL-6, TNF-alpha, and NF-kappa B p65 expression, worsening histopathological damage, and increased TUNEL and caspase-3 positivity indicate a similar pathophysiological pattern. However, because oxidative stress markers were not directly measured in this study, interpretations regarding oxidative stress–inflammation interactions should be considered indirect inferences based on the existing literature.

In animals exposed to social isolation stress, increases in serum transaminases, decreases in antioxidant capacity, and elevations in proinflammatory cytokines have been observed. In addition, social isolation may influence drug metabolism-related pathways (9). In the present model, increased TNF-alpha, NF-kappa B p65, and IL-6 levels, together with a concomitant rise in apoptosis markers, are consistent with the possibility that social isolation is associated with structural and molecular changes related to hepatic injury. It has also been reported that N-acetylcysteine administration after social isolation can regulate liver enzymes by suppressing interleukin-1 beta and IL-6 gene expression (17). In this regard, the partial improvement observed with vortioxetine suggests that isolation-related liver damage can be partially reversed by pharmacological interventions.

At the clinical level, large-scale prospective data have demonstrated that social isolation and loneliness increase the risk of nonalcoholic fatty liver disease (18). This parallel supports the notion that social isolation may be an independent risk factor for liver health. Depression has also been reported to be associated with nonalcoholic fatty liver disease risk, and this association appears to occur via the triglyceride-glucose index, representing the metabolic/insulin resistance axis, rather than via C-reactive protein (19). In the present model, marked tissue inflammation was observed, as indicated by IL-6, TNF-alpha, and NF-kappa B p65. In clinical studies, however, C-reactive protein, a systemic acute-phase marker, has been reported to be more limited in reflecting this relationship. This suggests that systemic and tissue-level inflammatory responses may differ in sensitivity and that the metabolic–insulin resistance axis may be more decisive in pathogenesis.

Accumulating evidence indicates that oxidative stress and proinflammatory pathways play central roles in the pathophysiology of depression (20, 21). Various experimental models support the notion that these processes are not limited to the central nervous system but may also affect peripheral organs. In chronic mild stress models, increases in reactive oxygen species and malondialdehyde levels in the liver, decreases in antioxidant enzyme activities, and accompanying histological alterations have been reported. Imipramine has been shown to only partially reverse this oxidative damage (22). Similarly, agomelatine treatment has been reported to reduce oxidative damage in brain, kidney, and liver tissues, although increases in plasma cytokine levels have also been described (23). Collectively, these reports suggest that oxidative stress may contribute to liver inflammation in depression models and that pharmacological effects may vary across tissues. In the present study, vortioxetine treatment was associated with reduced NF-kappa B p65, TNF-alpha, IL-6, and apoptosis marker levels. These findings may be related to the inflammatory and oxidative processes described in the literature; however, oxidative stress was not directly assessed in the present study.

The reciprocal interaction between the liver and brain suggests that peripheral inflammation may play a decisive role in neuroinflammation and behavioral responses. In a hepatic encephalopathy model, *Moringa oleifera* extract has been reported to reduce apoptosis and alleviate depressive behaviors by suppressing the Toll-like receptor 2/4-myeloid differentiation primary response 88-NF-kappa B pathways (24). Similarly, in a carbon tetrachloride model, matrine administration has been reported to improve behavioral deficits by reducing oxidative stress, neuroinflammation, and ammonia levels (25). In the present study, the reduction in inflammatory and apoptotic markers in liver tissue after vortioxetine treatment is consistent with these literature-based observations. However, vortioxetine may not exert a direct antioxidant effect. Any indirect influence may be related to neuroimmune interactions reported in previous studies, but this was not directly examined in the present study.

In the group receiving vortioxetine alone, liver histopathology and IL-6, TNF-alpha, and NF-kappa B p65 levels were comparable to those in the control group. In the SI+V group, the severity of these alterations was reduced compared with that in the SI group, although complete normalization was not observed. These findings are consistent with the generally favorable hepatic safety profile reported for vortioxetine in the literature. Possible cytochrome P450-related interactions have also been reported previously, particularly in polypharmacy settings, but such interactions were not evaluated in the present study. Therefore, these considerations should be interpreted cautiously. Although uncommon, antidepressant-induced liver injury represents a clinically relevant safety concern. Transient aminotransferase elevations or idiosyncratic drug-induced liver injury may be observed in a small percentage of patients (26, 27). Selective serotonin reuptake inhibitors generally have the lowest risk of hepatotoxicity, whereas agents such as agomelatine, mianserin, and clomipramine may carry a higher risk (28). This literature is consistent with the biochemical and histological findings observed in the group treated with vortioxetine alone, which were similar to those in the control group. The reduction in liver damage in the group treated with vortioxetine alongside social isolation may be associated with the reduced inflammatory marker expression observed in this study.

Finally, increased NF-kappa B p65 activation alongside increases in IL-6 and TNF-alpha was accompanied by evidence of apoptosis, as indicated by TUNEL and caspase-3 positivity. Although vortioxetine did not completely normalize these patterns, the observed reduction may support the involvement of oxidative stress-insulin resistance-inflammation interactions in liver damage in depression models (29). It has also been demonstrated in different models that the increase in apoptosis detected by caspase-3 immunoexpression and TUNEL positivity in liver tissue can be reduced by pharmacological interventions (30).

Decreased perivenular CK-18 staining suggests impaired hepatocyte structural integrity and a pattern compatible with hydropic degeneration/ballooning. This finding may be related to caspase-mediated cleavage of CK-18 during apoptosis. Previous studies have shown that CK-18 undergoes proteolytic degradation during apoptotic cell injury and may serve as a sensitive structural marker in early liver damage (31, 32). In the present study, reduced pan/total CK-18 staining was observed in the same regions in which TUNEL positivity and caspase-3 expression increased. This spatial association may support the presence of inflammation-related hepatocyte injury. The partial preservation observed in the SI+V group suggests attenuation of these changes, although no direct causal conclusion can be drawn.

Therefore, the present findings should be interpreted within the limitations of the assessed histopathological and molecular endpoints.

### 5.1. Limitations

This study has several limitations. First, behavioral outcomes related to the social isolation paradigm were not directly assessed, such as anhedonia- or stress-related behavioral tests, which limits the strength of inference regarding affective phenotypes in the present cohort. Oxidative stress markers and metabolic indicators, such as insulin signaling or lipid peroxidation, were not directly assessed, limiting detailed interpretation of the relationship between inflammatory and oxidative processes. Furthermore, molecular analyses were confined to immunohistochemistry and ELISA; therefore, the absence of confirmatory assays such as Western blot or gene expression profiling restricted the molecular depth of the data. Future comprehensive studies incorporating oxidative stress and metabolic parameters, hormonal responses, and behavioral readouts may provide a more integrated understanding of liver-brain interactions in depression.

### 5.2. Conclusions

The present findings indicate that social isolation is associated with increased hepatic inflammation, apoptosis, and histopathological alterations. Vortioxetine treatment was associated with partial attenuation of these parameters. These results are based on histological, immunohistochemical, and biochemical markers and should not be interpreted as direct evidence of functional hepatic protection or specific mechanistic pathways.

## Data Availability

The dataset presented in the study is available on request from the corresponding author during submission or after publication.

## References

[1] Murray CJ, Vos T, Lozano R, Naghavi M, Flaxman AD, Michaud C (2012). Disability-adjusted life years (DALYs) for 291 diseases and injuries in 21 regions, 1990 - 2010: a systematic analysis for the Global Burden of Disease Study 2010. Lancet.

[2] Chen XD, Li F, Zuo H, Zhu F (2025). Trends in prevalent cases and disability-adjusted life-years of depressive disorders worldwide: findings from the Global Burden of Disease Study from 1990 to 2021. Depress Anxiety.

[3] Grigoryan GA, Pavlova IV, Zaichenko MI (2022). Effects of social isolation on the development of anxiety and depression-like behavior in model experiments in animals. Neurosci Behav Physiol.

[4] Mumtaz F, Khan MI, Zubair M, Dehpour AR (2018). Neurobiology and consequences of social isolation stress in animal model-A comprehensive review. Biomed Pharmacother.

[5] Zhang M, Wu W, Huang C, Cai T, Zhao N, Liu S (2022). Shuxie-1 decoction alleviated CUMS-induced liver injury via IL-6/JAK2/STAT3 signaling. Front Pharmacol.

[6] Guo LT, Wang SQ, Su J, Xu LX, Ji ZY, Zhang RY (2019). Baicalin ameliorates neuroinflammation-induced depressive-like behavior through inhibition of toll-like receptor 4 expression via the PI3K/AKT/FoxO1 pathway. J Neuroinflammation.

[7] Li Q, Yang H, Wang W, Li N, Zou X, Li Y (2020). Brassica rapa polysaccharides ameliorate CCl4-induced acute liver injury in mice through inhibiting inflammatory apoptotic response and oxidative stress. Chem Biodivers.

[8] Xiao J, Lim LKE, Ng CH, Tan DJH, Lim WH, Ho CSH (2021). Is fatty liver associated with depression? A meta-analysis and systematic review on the prevalence, risk factors, and outcomes of depression and non-alcoholic fatty liver disease. Front Med (Lausanne).

[9] Zahir M, Shariatzadeh S, Khosravi A, Alshaikh FA, Moradi P, Ghaderi M (2021). High risk of drug toxicity in social isolation stress due to liver dysfunction: Role of oxidative stress and inflammation. Brain Behav.

[10] Bove M, Lama A, Schiavone S, Pirozzi C, Tucci P, Sikora V (2022). Social isolation triggers oxidative status and impairs systemic and hepatic insulin sensitivity in normoglycemic rats. Biomed Pharmacother.

[11] Sanchez C, Asin KE, Artigas F (2015). Vortioxetine, a novel antidepressant with multimodal activity: review of preclinical and clinical data. Pharmacol Ther.

[12] Barbosa-Méndez S, Perez-Sánchez G, Salazar-Juárez A (2022). Vortioxetine treatment decreases cocaine-induced locomotor sensitization in rats. Physiol Behav.

[13] Anwar MM, Laila IMI (2024). The ameliorating effect of rutin on hepatotoxicity and inflammation induced by the daily administration of vortioxetine in rats. BMC Complement Med Ther.

[14] Bétry C, Etiévant A, Pehrson A, Sánchez C, Haddjeri N (2015). Effect of the multimodal acting antidepressant vortioxetine on rat hippocampal plasticity and recognition memory. Prog Neuropsychopharmacol Biol Psychiatry.

[15] Pereda-Pérez I, Popović N, Otalora BB, Popović M, Madrid JA, Rol MA (2013). Long-term social isolation in the adulthood results in CA1 shrinkage and cognitive impairment. Neurobiol Learn Mem.

[16] Kesik V, Guven A, Vurucu S, Tunc T, Uysal B, Gundogdu G (2009). Melatonin and 1400 W ameliorate both intestinal and remote organ injury following mesenteric ischemia/reperfusion. J Surg Res.

[17] Asgharzadeh J, Derakhshan L, Asgharzadeh N, Mardani M, Shahrani D, Shahrani M (2025). N-acetylcysteine reduces the hepatic complications of social isolation stress through modulation of interleukin 1 and 6 gene expression and liver enzymes in mice. Sci Rep.

[18] Miao Y, Kong X, Zhao B, Fang F, Chai J, Huang J (2025). Loneliness and social isolation with risk of incident non-alcoholic fatty liver disease, UK biobank 2006 to 2022. Health Data Sci.

[19] Lee JW, Park SH (2021). Association between depression and nonalcoholic fatty liver disease: Contributions of insulin resistance and inflammation. J Affect Disord.

[20] Huang X, Liu X, Yu Y (2017). Depression and chronic liver diseases: are there shared underlying mechanisms?. Front Mol Neurosci.

[21] Bhatt S, Nagappa AN, Patil CR (2020). Role of oxidative stress in depression. Drug Discov Today.

[22] Duda W, Curzytek K, Kubera M, Iciek M, Kowalczyk-Pachel D, Bilska-Wilkosz A (2016). The effect of chronic mild stress and imipramine on the markers of oxidative stress and antioxidant system in rat liver. Neurotox Res.

[23] Demirdaş A, Nazıroğlu M, Ünal GÖ (2016). Agomelatine reduces brain, kidney and liver oxidative stress but increases plasma cytokine production in the rats with chronic mild stress-induced depression. Metab Brain Dis.

[24] Mahmoud MS, El-kott AF, AlGwaiz HIM, Fathy SM (2022). Protective effect of Moringa oleifera Lam. leaf extract against oxidative stress, inflammation, depression, and apoptosis in a mouse model of hepatic encephalopathy. Environ Sci Pollut Res Int.

[25] Khan A, Shal B, Naveed M, Shah FA, Atiq A, Khan NU (2019). Matrine ameliorates anxiety and depression-like behaviour by targeting hyperammonemia-induced neuroinflammation and oxidative stress in CCl4 model of liver injury. Neurotoxicology.

[26] Park S, Ishino R (2013). Liver injury associated with antidepressants. Curr Drug Saf.

[27] Voican CS, Corruble E, Naveau S, Perlemuter G (2014). Antidepressant-induced liver injury: a review for clinicians. Am J Psychiatry.

[28] Friedrich ME, Akimova E, Huf W, Konstantinidis A, Papageorgiou K, Winkler D (2016). Drug-induced liver injury during antidepressant treatment: results of AMSP, a drug surveillance program. Int J Neuropsychopharmacol.

[29] Zong Y, Chen T, Dong H, Zhu L, Ju W (2020). Si-Ni-San prevents reserpine-induced depression by inhibiting inflammation and regulating CYP450 enzymatic activity. Front Pharmacol.

[30] Kortam MA, Ali BM, Fathy N (2021). The deleterious effect of stress-induced depression on rat liver: Protective role of resveratrol and dimethyl fumarate via inhibiting the MAPK/ERK/JNK pathway. J Biochem Mol Toxicol.

[31] Hetz H, Hoetzenecker K, Hacker S, Faybik P, Pollreisz A, Moser B (2007). Caspase-cleaved cytokeratin 18 and 20 S proteasome in liver degeneration. J Clin Lab Anal.

[32] Yilmaz Y (2009). Systematic review: caspase-cleaved fragments of cytokeratin 18-the promises and challenges of a biomarker for chronic liver disease. Aliment Pharmacol Ther.

